# Recent Developments in Electrospun Nanofiber-Based Triboelectric Nanogenerators: Materials, Structure, and Applications

**DOI:** 10.3390/membranes14120271

**Published:** 2024-12-16

**Authors:** Qinglong Wei, Yuying Cao, Xiao Yang, Guosong Jiao, Xiaowen Qi, Guilin Wen

**Affiliations:** School of Mechanical Engineering, Yanshan University, Qinhuangdao 066004, Chinayxiao.ysu@hotmail.com (X.Y.);

**Keywords:** electrospinning, TENG, materials, structure design, application

## Abstract

Triboelectric nanogenerators (TENGs) have garnered significant attention due to their high energy conversion efficiency and extensive application potential in energy harvesting and self-powered devices. Recent advancements in electrospun nanofibers, attributed to their outstanding mechanical properties and tailored surface characteristics, have meant that they can be used as a critical material for enhancing TENGs performance. This review provides a comprehensive overview of the developments in electrospun nanofiber-based TENGs. It begins with an exploration of the fundamental principles behind electrospinning and triboelectricity, followed by a detailed examination of the application and performance of various polymer materials, including poly (vinylidene fluoride) (PVDF), polyamide (PA), thermoplastic polyurethane (TPU), polyacrylonitrile (PAN), and other significant polymers. Furthermore, this review analyzes the influence of diverse structural designs—such as fiber architectures, bionic configurations, and multilayer structures—on the performance of TENGs. Applications across self-powered devices, environmental energy harvesting, and wearable technologies are discussed. The review concludes by highlighting current challenges and outlining future research directions, offering valuable insights for researchers and engineers in the field.

## 1. Introduction

As global energy demand continues to rise and traditional energy resources gradually deplete, the quest for efficient, environmentally friendly, and renewable energy solutions has become a major focus of scientific research and technological development [1,2,3]. The rapid increase in energy consumption and growing environmental challenges compel us to seek new energy technologies that can reduce dependence on finite resources [4,5,6,7].

Triboelectric nanogenerators (TENGs), with their unique operating principles and significant performance advantages, are emerging as crucial technologies in energy harvesting and management [8,9,10,11,12,13]. TENGs convert mechanical energy into electrical energy through friction and contact electrification, providing an innovative solution to address energy shortages and environmental pollution challenges [14,15,16,17]. Recent advancements in electrospun nanofiber technology have shown immense potential to enhance TENGs performance and broaden their application modalities [18,19,20,21].

Electrospun nanofibers are particularly promising for TENGs applications due to their exceptional specific surface area, high porosity, and favorable mechanical properties [22,23,24,25]. The electrospinning technique enables the fabrication of nanoscale fibers that provide a vast surface contact area, enhancing the efficiency of mechanical energy capture and conversion during friction processes [7,26,27,28]. By adjusting the spinning solutions of different polymers, as well as fiber alignment and structural design, electrospun nanofibers can significantly improve the energy conversion efficiency and stability of TENGs. Moreover, their flexibility and tunability allow for diverse designs of TENG structures, driving advancements in the technology across various application scenarios [29,30].

Although electrospun nanofiber-based TENGs have been extensively discussed in the literature, several crucial aspects, such as the selection of materials for the positive and negative triboelectric layers, the exploration of various structural designs, and the expansion of their applications, have not been thoroughly, systematically, or uniformly reviewed. This review aims to systematically assess the current research status and progress of electrospun nanofiber-based TENGs. In comparison with previous reviews, we first elucidate the fundamental principles of electrospinning and TENGs, emphasizing the pivotal role of electrospinning in enhancing TENGs performance. We then provide an in-depth analysis of the polymer materials relevant to the positive and negative triboelectric layers, followed by a detailed examination of diverse structural designs. Furthermore, this review explores various application scenarios, including self-powered devices, environmental energy harvesting, and wearable technologies. We also address the current challenges and future development directions, offering comprehensive references and insightful perspectives to researchers in the field, thereby promoting the further application and advancement of electrospun nanofibers in TENGs technology.

## 2. Basic Foundation of Electrospun Nanofiber-Based TENGs

Electrospinning is a widely utilized technique for producing nanofibers, which involves the application of a high voltage to a polymer solution. This creates an electrostatic field that elongates the fibers into fine, elongated strands. In contrast, the triboelectric effect refers to the phenomenon where static charges are generated through friction between two materials. The integration of electrospinning and the triboelectric effect enables the development of TENGs, which have extensive applications in energy harvesting and sensors. This section provides a detailed overview of the principles of electrospinning and the triboelectric effect.

### 2.1. Principle of Electrospun Nanofibers

Electrospinning is a crucial technique for fabricating nanofibers. In a typical setup, a polymer solution is extruded through a spinneret, forming droplets at a specific concentration. Under the influence of a high electric field, these droplets at the needle’s tip reshape from a spherical form to a conical shape, known as the “Taylor cone”, due to electrostatic repulsion between like surface charges [31,32,33]. When the voltage reaches a critical level, the electric field force overcomes the liquid’s surface tension, causing a jet to emerge from the Taylor cone.

**Figure 1:**
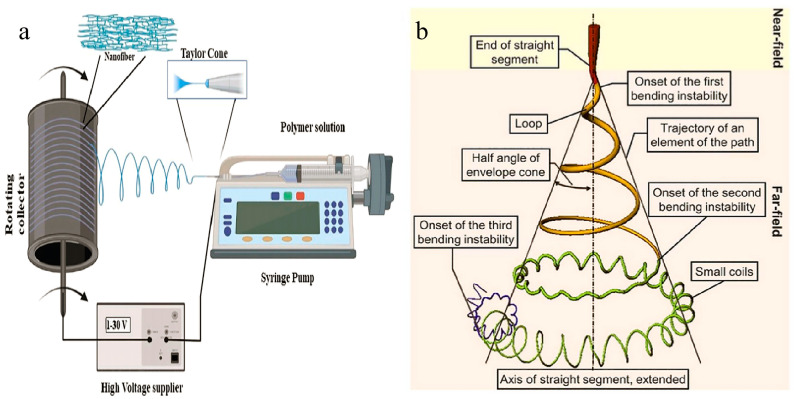
Principle of electrospun nanofibers. (**a**) Schematic diagram of a basic electrospinning setup [34]. (**b**) Schematic diagram showing the path of an electrospun jet [35]. All essential copyrights and permissions received.

In this high electric field, the jet experiences unstable oscillations and high-frequency spiral movements. As it oscillates quickly, the jet is stretched, the solvent evaporates rapidly, and nanometer-sized fibers are formed. These fibers then deposit randomly onto a collector, resulting in a nonwoven fabric [34]. As shown in Figure 1a, it is the basic setup diagram of electrospinning. Between the syringe pump and the collector, there is an unstable region where the polymer is stretched into nanofibers, which is represented by the blue line in Figure 1a.

In essence, the electrospinning process involves droplet charging, Taylor cone formation, jet stretching, and thinning, followed by the solidification and collection of the nanofibers (Figure 1b) [35]. The nanofibers produced exhibit a random network structure, uniform diameter, smooth surface, and discrete fiber connections [36]. The electrospinning technique is user-friendly and cost-effective. It is important to note that multiple factors influence the process, including polymer properties (e.g., molecular weight), solution concentration, solvent characteristics, voltage, flow rate, and the distance between the needle and the collector [37,38,39,40,41].

### 2.2. Principle of Triboelectricity

The triboelectric effect occurs when two different materials come into contact and are rubbed together, resulting in the transfer of electrons from one material to the other. This electron transfer leads to one material becoming negatively charged while the other becomes positively charged. In TENGs design, materials are chosen based on their position in the triboelectric series to maximize charge transfer [20,42].

In a TENGs device, the charged materials establish an electric field. Mechanical movements such as compression, friction, or sliding facilitate continuous contact and separation between the material surfaces, leading to periodic charge transfer between the electrodes. This variation in charge generates alternating voltage and current in the circuit, thus producing electrical energy [42,43,44].

**Figure 2:**
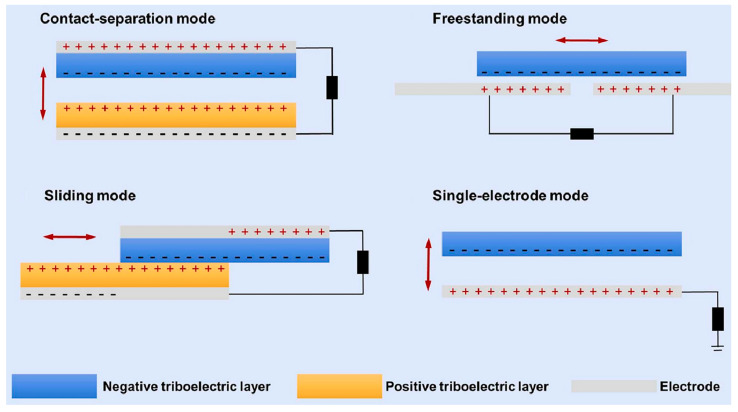
Working modes of TENGs (the red arrows indicate the direction of triboelectric layers movement; +: positive charge; −: negative charge) [24]. All essential copyrights and permissions received.

TENGs operate in four basic modes: contact-separation mode, sliding mode, freestanding mode, and single-electrode mode (Figure 2) [24]. The most prevalent mode in electrospun nanofiber-based TENGs is the contact-separation mode [45,46,47,48]. In this mode, the periodic contact and separation of different materials induce charge transfer and alter the electric field, resulting in alternating voltage and current between the electrodes, thereby achieving efficient energy conversion [49,50,51,52,53].

In summary, electrospinning technology produces nanofibers with a high specific surface area, significantly increasing the friction surface and improving charge generation and transfer efficiency in TENGs [54]. Moreover, electrospinning allows precise control over fiber diameter and structure, optimizing TENGs performance [55]. These nanofibers also exhibit excellent mechanical properties, chemical stability, and flexibility, enabling adaptability to various surfaces and shapes, ultimately enhancing the overall efficiency of TENGs [56,57,58].

## 3. Materials for Nanofiber-Based Triboelectric Nanogenerators

The choice of materials plays a crucial role in the effectiveness of nanofiber-based TENGs. Materials like polyvinylidene fluoride (PVDF) and polyacrylonitrile (PAN) are commonly selected for their superior flexibility and electrical properties, which are vital for TENG functionality [59,60]. The selection of these materials significantly influences the output performance of TENGs, particularly in terms of voltage and current generation. In this section, we examine the materials typically employed in the construction of nanofiber-based TENGs.

### 3.1. Positive Triboelectric Layer Materials for Nanofiber-Based TENGs

The charges in TENGs are generated through the contact and separation between two triboelectric (TE) layers with different electron affinities, making the proper selection and fabrication of TE materials crucial for achieving high-output TENGs [61]. Polyamide (PA, Nylon), with its high positive electron affinity and strong electron-donating characteristics, has become an ideal choice for preparing positive triboelectric materials in TENGs. Currently, most researchers have improved the performance of polyamide-based positive triboelectric materials through strategies such as surface coating, cationic functionalization, nanocomposite preparation, and electric field regulation [62,63,64,65]. For example, in Huang et al., a simple, scalable, and one-pot electrospinning fabrication technique was utilized to construct an all-fiber-structured triboelectric nanogenerator (TENG) [66]. The assembled all-fiber TENG exhibited excellent durability and stability, as well as excellent output performance, which reached a peak power density of 290 mW/m^2^ at a load resistance of 100 MΩ. Yar et al. incorporated AgSbS_2_ nanocrystals with high surface roughness into a polyamide polymer and fabricated AgSbS_2_@Nylon 6.6 nanofiber mats using electrospinning technology [67]. This approach increased the effective contact area, electron dissipation capability, and surface roughness of the positive dielectric material, thereby enhancing the performance of the TENG. For the 10 wt% AgSbS_2_@Nylon 6.6 nanofiber-based TENG, the maximum output voltage, power density, and capacity voltage were found to be 546 V, 6.81 W/m^2^, and 10 V, respectively. Additionally, Choi et al. prepared a composite nanofiber (NF) structure of nylon 66 and mica, which exhibits excellent electrical insulation, low dielectric loss, high thermal conductivity, and enhanced triboelectric performance to address these issues [68]. Incorporating mica into the nylon NFs improved TENG performance, preventing performance degradation even in harsh environments with a relative humidity of 70%. In another study, Prasad et al. developed a simple post-surface modification technique using poly-L-lysine (PLL), which, for the first time, enhanced the positive polarity of Nylon 11 electrospun membranes [69]. The performance of the PLL-modified Nylon 11 electrospun membranes (PNy11) as a positive electrode layer was compared with that of the unmodified Nylon 11 membranes (Ny11). The frictional electrical output performance of PNyl 11 was significantly enhanced, with the open-circuit voltage increasing by over five times (from 26 V to 137 V) and the short-circuit current rising more than fourfold (from 0.8 µA to 3.4 µA). In summary, the unique properties of nylon make it a commonly used positive friction layer in TENGs [70,71,72].

Moreover, thermoplastic polyurethane (TPU) is a highly esteemed typical polymer among general materials, renowned for its exceptional mechanical toughness, chemical elasticity, biocompatibility, and flexibility [73,74]. Notably, TPU also inherently possesses the capacity to release triboelectric charges [16]. Consequently, some researchers have capitalized on these attributes and employed it as a positive triboelectric layer in the construction of TENGs by means of electrospinning technology. For example, Yan et al. developed a flexible triboelectric layer, where an electrospun ethylcellulose (EC)/TPU nanofiber membrane provided a high-roughness triboelectric surface. Additionally, barium titanate (BTO) nanoparticles were incorporated to enhance the output performance through the synergistic effects of piezoelectricity and triboelectricity [74]. The resulting TENGs exhibited superior triboelectric performance compared to both individual piezoelectric nanogenerators and TENGs, as well as remarkable durability and stability. Li et al. designed a high-performance triboelectric nanogenerator (TENG) based on TPU/mica nanofibers [75]. They paired TPU/mica nanofibers with PVDF/MXene nanofibers. As the concentration of mica nanosheets increased to 7.5 wt%, the transferred charge of the TENG increased from 38.6 nC to 82.4 nC. However, with further increases in mica concentration, the transferred charge decreased.

PAN, one of the most commonly used polymers in electrospinning processes, is also frequently utilized as the positive triboelectric layer in TENGs due to its relatively high dielectric constant [13,76]. Yar et al. first prepared flexible nanofibers of PAN/ZnO and PAN/B(OH)_3_ as triboelectric contact layers to enhance the power generation performance of PAN [77]. At a load of 33 MΩ, the peak power density of a 3 cm × 3 cm PAN/B(OH)_3_ structure reached 6.67 W/m^2^. Due to the limited crystallinity of ZnO synthesized from solution, no significant improvement in the power generation performance of PAN was observed. In another study, Kinas et al. prepared a spring-supported triboelectric nanogenerator (TENG) consisting of polyvinylpyrrolidone/ethyl cellulose (PVP/EC) nanofibers and various carbon-doped PAN nanofibers as the positive and negative dielectric layers, respectively [78]. Experimental results showed that grafting reduced graphene oxide (rGO) and carbon nanotubes (CNTs) onto the PAN matrix significantly increased the surface charge density of the TENG and improved its output voltage.

In addition to the aforementioned positive triboelectric layer materials, Sardana et al. synthesized an electrospun triboelectric nanogenerator (TENG) by pairing highly electronegative and conductive MXene nanofibers with biodegradable cellulose acetate (CA) nanofibers as the triboelectric layer, with CA nanofibers serving as the positive triboelectric layer material [79]. This TENG achieved a sufficient power density (~1361 mW/m^2^ at 2 MΩ) and demonstrated self-powering capability to operate a chemical resistance gas sensor manufactured in their work. Jo et al. fabricated a lignin/polycaprolactone nanofiber (NF)-based TENG using an electrospinning technique [80]. Here, the lignin/polycaprolactone NF served as the positive triboelectric layer. The output voltage of the lignin-based TENG exceeded 95 V, even under a relatively low tapping force of 9 N and a frequency of 9 Hz. Wang et al. innovatively selected the biocompatible poly (lactide-co-caprolactone) (PLCL) to modify the properties of the polyhydroxybutyrate (PHB) electrospun membranes and constructed PHB/PLCL composite membranes with different contents of PLCL [81]. The PHB/PLCL membranes served as the positive triboelectric layer and formed the TENG together with expanded polytetrafluoroethylene (ePTFE) membranes. Compared with the TENG composed of pure PHB membranes, the short-circuit voltage, short-circuit current, and short-circuit transferred charge density of the TENG composed of PHB/PLCL membranes with a PLCL content of 10% were increased by 38.89%, 63.50%, and 72.18%, respectively. In addition, nickel–copper and polyvinyl butyral decorated with graphene nanoplatelets (GNPs) and tetraethyl orthosilicate (TEOS) can be used as the positive triboelectric layer [82,83].

The latest research on positive triboelectric layer materials for nanofiber-based TENGs is summarized in Table 1.

### 3.2. Negative Triboelectric Layer Materials for Nanofiber-Based TENGs

PVDF nanofibers, with their unique piezoelectric properties, high specific surface area, flexibility, processability, and biocompatibility, are excellent negative triboelectric layer materials for TENGs [84,85,86,87,88]. Currently, many researchers are incorporating fillers into PVDF nanofibers to enhance their piezoelectric and dielectric properties, thereby further improving the performance of triboelectric nanogenerators [47,89,90,91,92]. For example, Zhang et al. designed a ternary coupling effect of a triboelectric–piezoelectric hybrid nanogenerator (T-PENG) based on the nanoporous film of PVDF/BTO composite nanofibers prepared by electrospinning [93]. At the optimum BTO content, the transfer charge density of the nanoporous T-PENG was 2.12 times the sum of the transfer charge density for the corresponding nanoporous PENG and pristine nanoporous TENG. Under the impact force of 15 N, the output voltage, current density, and transfer charge density of the optimized T-PENG were as high as 444 V, 19.02 mA/m^2^, and 105.6 μC/m^2^. Bai et al. successfully fabricated a TENG using electrospun NFs based on PVDF-aliphatic HBP of first-generation (PVDF/HBP-G1) blend NFs [48]. Moreover, among the combinations of PVDF/HBP-G1, the 10 wt%-based TENG (PA10/Al-TENG) had an output current of 1.76 μA (2 times that of PVDF(PA0)). In Rahman et al., for the first time, metal–organic framework (MOF)-derived cobalt-based nanoporous carbon (Co-NPC) particles were introduced into a PVDF matrix as nanofillers, followed by an electrospinning process to fabricate Co-NPC/PVDF composite NFs for a high-performance TENG [94]. The addition of Co-NPC up to the optimum ratio (0.5 wt%) increased the dielectric constant, surface potentials, and charge trapping capability of the Co-NPC/PVDF NFs by 2.75, 4, and 9.5 times, respectively, which synergistically enhanced the performance of CNP-TENG. In Bhatta et al., electrospun MXene (Ti3C2Tx)-functionalized PVDF composite nanofiber was firstly proposed as a promising negative triboelectric layer for boosting triboelectric energy harvesting performance [95]. The TENG could deliver a peak power of 4.6 mW (power density of 11.213 W/m^2^) at the matching impedances of 2 MΩ and exhibited excellent outputs even at very low frequency and low force impact motions. In Banerjee et al., Zn- and Sn-doped potassium sodium niobate (KNN-ZS) nanorods were first synthesized through a hydrothermal process. Afterwards, the doped KNN-ZS samples were used to prepare a PVDF/KNN-ZS nanocomposite fibrous web through the process of electrospinning, which was finally assembled into a TENG [96]. The TENG output voltage was observed as 25 V and its current was ∼2.11 μA. In addition, materials such as nickel oxide nanoparticles (NiO NPs), polystyrene (PS), zinc oxide nanowires (ZnO NWs), and fullerene (C_60_) can be used as fillers and incorporated into electrospun PVDF nanocomposites to enhance the performance of triboelectric nanogenerators (TENGs) based on PVDF nanofiber films [51,52,53,97,98,99,100,101,102,103].

In addition to PVDF, its copolymers, including PVDF-TrFE [104,105,106], poly (hydroxybutyrate-co-hydroxyvalerate) (PVDF-HFP) [107,108,109], have been widely investigated and applied as negative triboelectric layers for TENGs. For instance, in Xi et al., a high-performance TENG was fabricated based on BaTiO_3_:La-embedded PVDF-TrFE nanofiber membrane (NM) (BLPT-NM) for energy harvesting and wireless power transmission [110]. The TENG demonstrated excellent output performance with a power density of 2.52 W/m^2^ (ƒ = 1.5 Hz) and a triboelectric charge density of 87.3 μC/m^2^, which were significantly increased by more than 11- and 3-fold, respectively, in comparison with those of the TENG based on pristine PVDF-TrFE-NM. In another study, Deswal et al. designed an electrospun-based TENG comprising molecular ferroelectric, diisopropylammonium bromide (DIPAB)/PVDF-TrFE as an active negative layer [111]. The synergistic effects emanating from highly aligned polymeric chains and ferroelectric particles in conjunction with a high surface area of the as-designed TENG generated an output voltage of 203.8 V and resulted in a maximum power density of 416.2 mW/m^2^ when operated in contact-separation mode. In addition, Lin et al. were the first to report a nanofiber-based TENG fabricated from a simple blend of the organic semiconductive polymer poly (3-hexylthiophene) (P3HT), used to enhance the electrical output properties of the device [112]. The maximum output voltage of the P3HT/PVDF-HFP nanofiber TENG device reached up to 78 V with a corresponding output current of 7 μA under a cyclic compressive force of 30 N applied at a frequency of 5 Hz. The maximum output power that could be obtained was 0.55 mW, sufficient to power 500 red light-emitting diodes (LEDs) instantaneously. Sha et al. presented a technique for introducing liquid metal (LM) Galinstan nanodroplets into electrospun PVDF-HFP nanofibers to enhance their triboelectric performance [113]. Using the PVDF-HFP/2%LM nanofiber membrane as the negative tribo-layer and TPU as the positive tribo-layer, the peak open-circuit voltage and power density of the resultant TENG reached 1680 V and 24 W/m^2^, respectively, which were significantly higher than previous state-of-the-art values of existing PVDF-based TENGs.

These innovative composite nanofibers based on PVDF and its copolymers, integrating functional nanofillers, have not only enhanced TENGs output performance but also facilitated their adaptation to various application fields.

Apart from PVDF and its copolymers that can serve as negative triboelectric layers for electrospinning-based TENGs, other special materials can also be used as negative triboelectric layers [3,17,114]. For example, Yan et al. synthesized carbon nanotube@barium titanate (CNT@BTO) nanoparticles by the chemical vapor deposition method, and then incorporated them into a flexible polyimide (PI) negative triboelectric layer, which was prepared using electrospinning technology and thermal imidization [115]. This was combined with AL to form a TENGs. When the BTO content was 26 wt% and the CNT content was 2 wt%, the open-circuit voltage and short-circuit current of the TENG based on the PI/CNT@BTO nanofiber membrane reached 305 V and 104 μA, which were three times higher than those of the pure PI-based TENG. Xie et al. utilized [CNTs/PVDF/PVP]//[Eu(TTA)_3_(TPPO)_2_/PVDF/PVP] Janus nanofiber (JNM) as the negative triboelectric layer, which was combined with Poly (methyl methacrylate) (PMMA) to form a TENG [116]. This TENG was capable of achieving a high output performance, with the maximum output performance being 22.4 μA, 353.5 V, and 135.5 μC/m^2^.

Table 2 comprehensively summarizes the recent research outcomes of negative triboelectric layer materials for nanofiber-based TENGs.

In summary, the electrospinning of various polymers enables the production of nanofibers with different properties, which can be used to create a variety of TENGs. The appropriate selection of materials enhances the output performance and efficiency of TENGs based on electrospun nanofibers. However, researchers have predominantly used polymers like PVDF, PAN, nylon, and their derivatives and composites, which have been extensively studied. There is a pressing need to incorporate more novel polymer materials into electrospun nanofiber-based TENGs to further advance their capabilities.

## 4. Structure Design

Beyond the selection of materials, the structural design of electrospun fiber-based TENGs plays a pivotal role in their overall performance. The meticulous design of the fiber structure is essential to enhance TENGs efficiency, involving the optimization of the fibers themselves, the adoption of biomimetic structures, and the construction of multilayered configurations. These design strategies significantly improve the performance by increasing surface friction, enhancing charge transfer efficiency, and raising energy density. In this section, we provide a comprehensive discussion on these three aspects of structural design.

### 4.1. Fiber Structure

Nanofibers, owing to their high specific surface area and adjustable physical properties, profoundly impact friction and charge transfer efficiency [54,60,117]. Modifying factors such as fiber diameter, arrangement, and surface structure can increase the contact area for charge transfer, thereby enhancing mechanical strength and stability while improving charge accumulation efficiency and overall TENG performance [59,118,119,120,121].

**Figure 3:**
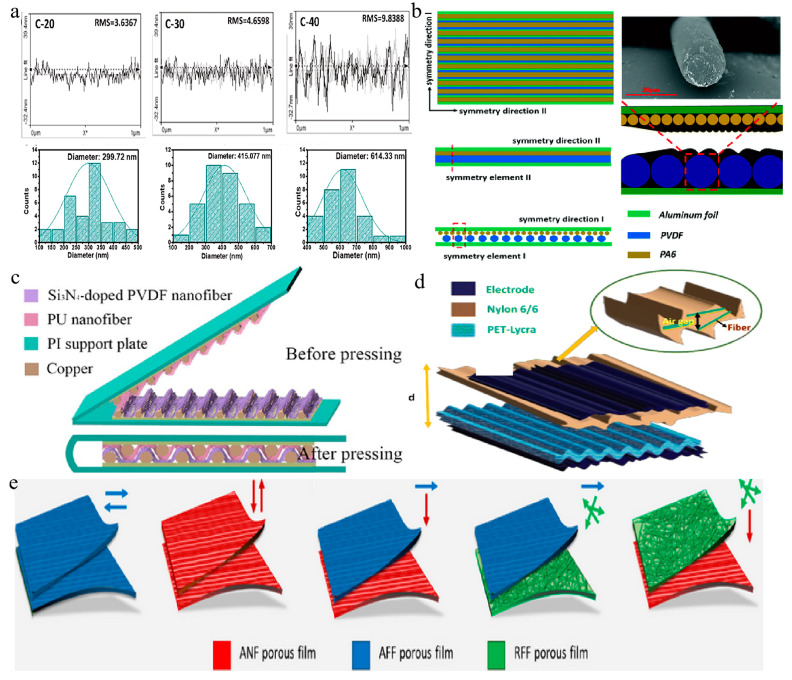
Fiber structure. (**a**) Surface roughness curves and fiber diameter histograms of electrospun fiber membranes at different humidity levels [122]. (**b**) Schematic diagram of the grating TENG, including top view, side view, and cross-sectional view, as well as a fiber cross-sectional view of PVDF (red dash lines denote the top view, side view, cross-sectional view and SEM images of the same sample position) [123]. (**c**) Wave-shaped TENG [127]. (**d**) Wrinkle-type TENG (yellow arrow indicates the distance between the upper and lower layers) [128]. (**e**) Stack configuration of electrospun PVDF with different dipole orientation and direction [130]. All essential copyrights and permissions received.

For instance, Ishu et al. employed electrospinning to precisely regulate nanofiber diameter by adjusting humidity, thereby influencing the surface morphology, roughness, and friction contact area of the triboelectric materials (see Figure 3a) [122]. This led to an augmented surface charge density, intensifying the generation of surface charges on the triboelectric materials. In comparison to CA-20_MXene (MX)-20, the optimized sample CA-40_MX-40 at 40% RH, effectively pairing MX and CA, resulted in a fivefold increase in voltage, achieving a peak power density of 2351.1 mW/m^2^ at a load of 10^6^ Ω, capable of powering an LED. In another study, Zhou et al. fabricated an ordered PVDF fiber membrane using a near-field electrospinning device and an ordered PA6 membrane using a parallel electrode collection technique, and then assembled them into a grating TENG (see Figure 3b) [123]. The resultant TENG produced a current of 870 nA and a voltage of 228 V, sufficient to power 230 LEDs without an additional energy storage device. Furthermore, Li et al. examined the influence of various fiber arrangements during the electrospinning process on triboelectric nanogenerator performance [124].

The deliberate design of fiber surface structure can also heighten TENG performance [125,126]. Zhou et al. synthesized a TENG with a microwaved-shaped structure (MW-EPTENG) using electrospinning (see Figure 3c) [127]. The MW-EPTENG demonstrated a peak output voltage of 102 V and a current of 1.02 μA, significantly surpassing that of planar-structure triboelectric nanogenerators with polarized nanofibers. Notably, the MW-EPTENG maintained a stable output current and voltage even after continuous compression for 6 h and illuminated a 21.5 mm diameter LED bulb. In another study, Haghayegh et al. enhanced the stretchability of TENG components by utilizing structured wrinkled nylon 6/6 nanofibers as the triboelectric layer, wrinkled poly (3,4-ethylenedioxythiophene)/poly (styrenesulfonate) (PEDOT:PSS) as the electrode, and wrinkled polyethylene terephthalate (PET)/spandex fabric (Lycra)/silver nanowire–single-walled carbon nanotubes (AgNW-SWCNTs) as the triboelectric electrode layer (see Figure 3d) [128]. The resulting textile-based wrinkled stretchable TENG (WS-TENG) achieved an instantaneous peak power density of 10.23 mW/cm^2^ under a resistance of 10^9^ Ω, with no significant variation in open-circuit voltage during continuous pressing and releasing, signifying excellent robustness and stability.

Furthermore, augmenting the output performance of TENG can be achieved by introducing dipoles between the fibers [129]. Rastegardoost et al. developed a high-performance TENG using porous PVDF mats, featuring enhanced dielectric properties and a novel dipole arrangement [130]. Through the control of various process parameters, they produced single-layer electrospun mats with Aligned Near-Field (ANF), Aligned Far-Field (AFF), and Random Far-Field (RFF) configurations. The alignment of dipoles significantly enhanced the dielectric constant of the electrospun mats. When stacked in various smart multilayer configurations, the electrospun porous mats with oriented dipoles exhibited a notably high dielectric constant of up to 10, comparable to values obtained from nonporous original PVDF films (see Figure 3e). This novel configuration, combined with nylon as the counter dielectric, was integrated into a curved TENG device, achieving an output voltage exceeding 130 V and a current of up to 12 μA, significantly higher than that of nonporous original PVDF films and single-layer electrospun mats. At an optimal external resistance of approximately 20 MΩ, the maximum power density reached 3.5 W/m^2^.

In summary, optimizing the internal structure of fibers leads to a significant improvement in charge generation and transfer efficiency, thereby enhancing the performance of the material in energy harvesting applications. However, this optimization process also has some inherent limitations. The design and creation of complex internal structures in fibers often require sophisticated manufacturing techniques, which can complicate the production process, increase costs, and may result in lower production yields or higher defect rates. Additionally, the alterations to the fibers might affect their mechanical properties, potentially weakening tensile strength, flexibility, or durability, thus changing their original characteristics.

### 4.2. Bionic Structure

Special fiber network architectures can significantly boost material utilization and performance. In the realm of TENG technology, the use of biomimetic nanofibers holds particular significance. Researchers frequently draw inspiration from natural biological nano/microstructures, leveraging electrospinning parameters and material composition adjustments to generate highly controllable and customizable nanofibers [131,132]. This approach allows for the precise tailoring of nanofiber dimensions, shapes, porosity, and properties, satisfying specific application requirements and achieving tailored performance and functionality.

**Figure 4:**
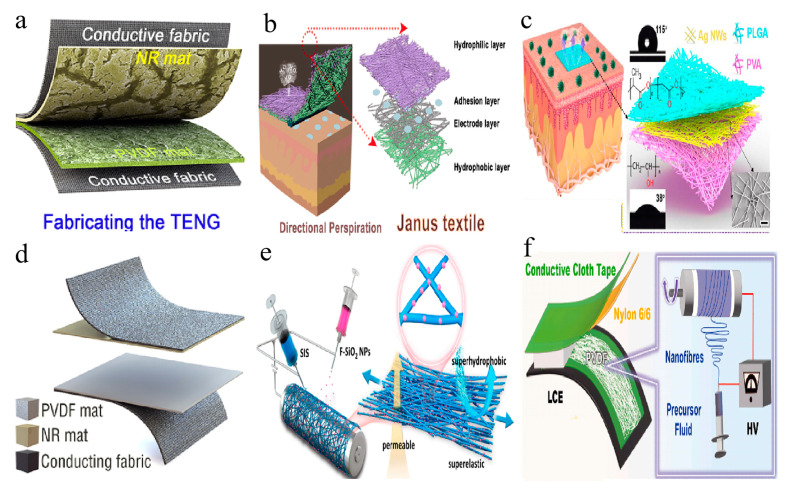
Bionic structure. (**a**) TENG based on petiole-shaped fiber mat [136]. (**b**) Janus textile inspired by the internal structures of plants (red dot lines mark a small part of the Janus textile that will be attached to the skin and point to the corresponding structure) [137]. (**c**) Structural design of the TENG-based e-skin (black dot lines mark the all-nanofiber TENG-based e-skin and point to the corresponding structure.) [141]. (**d**) Bio-inspired hydrophobic/cancellous/hydrophilic Trimurti-based TENG [142]. (**e**) Silk-inspired nanofibers [143]. (**f**) Bioinspired soft TENG fabricated based on animal body structures [144]. All essential copyrights and permissions received.

Inspired by plant fiber networks, researchers have developed a series of biomimetic nanofiber structures [133,134]. These structures efficiently utilize materials and significantly enhance the energy conversion efficiency of TENGs, bestowing them with unique performance characteristics [135]. For instance, Zhang et al., drawing inspiration from plants, devised a biomimetic petiole-shaped microfiber-based friction material with internal nanocavities, a rough surface, and superhydrophobicity using a simple, low-waste, and efficient single-component electrospinning process [136]. The petiole-shaped structure and superhydrophobicity equipped the assembled triboelectric nanogenerator (PMF-TENG) with outstanding electrical performance and excellent output stability under humid conditions (see Figure 4a). The optimized PMF-TENG demonstrated a high power density of 56.9 W/m^2^ and a peak output voltage of 2209 V. At 80% relative humidity, the output retention rate of the optimized PMF-TENG was 1.7 times and 2.2 times higher compared to TENGs made with conventional smoother solid nanofiber-based friction materials and single-layer nanoporous friction materials, respectively. Furthermore, taking inspiration from the internal structures of plants used for water transport, Cheng et al. developed a fully nanofiber Janus textile using continuous electrospinning/electrospraying techniques [137]. This textile exhibited dual-gradient variations in pore size and wettability along its thickness direction, enabling directional sweat transport (anti-gravity delivery) performance (see Figure 4b). As a mechanical energy harvester, the TPU layer served as the triboelectric layer, achieving a maximum open-circuit voltage (Voc) of 78.10 V, a short-circuit current (Isc) of 0.16 μA, and a power density of 3.31 W/m^2^.

The skin, as one of the largest sensory organs in the human body, excels at detecting various external stimuli such as temperature, pressure, and touch. This unique capability provides valuable inspiration for designing nanofiber membranes with skin-like sensory functions [138,139,140]. Inspired by the skin, Peng and colleagues designed a breathable, biodegradable, and antibacterial electronic skin based on a fully nanofiber triboelectric nanogenerator (TENG) [141]. This electronic skin (e-skin) was crafted by embedding silver nanowires (Ag NWs) between poly (lactic acid)-glycolic acid (PLGA) and PVA (see Figure 4c). With its micro-nano hierarchical porous structure, the electronic skin featured a high specific surface area, facilitating effective contact with charged objects and providing numerous capillary channels for thermal and moisture transfer. The single-electrode mode TENG e-skin demonstrated a maximum peak power density of 130 mW/m^2^ and a voltage response pressure sensitivity of 0.011 kPa^−1^, enabling it to monitor various physiological signals such as blinking, pulse, speaking, and breathing.

Additionally, Zhang and colleagues designed a bio-inspired Trimurti PVDF tribo-material through a simple self-assembly process involving electro-pore creation [142]. This material displayed superb electrical properties, outstanding output stability in high ambient humidity, and enhanced comfort under perspiration conditions. The nano-porous cancellous bone-like, hydrophobic lotus leaf-like, and hydrophilic root-xylem-like structures were assembled on the inside, upper surface, and underside of the Trimurti PVDF felt, respectively (see Figure 4d). The fabricated Trimurti triboelectric nanogenerator (T-TENG) demonstrated superior electrical performance and usability in various application environments. With a high power density of 10.6 W/m^2^, the T-TENG could directly power 714 LEDs and small electronic devices. Furthermore, at 85% relative humidity, the T-TENG maintained an output retention rate of up to 22%. Under simulated sweating conditions, the Trimurti PVDF pad could absorb sweat into its bottom area, accelerating sweat evaporation.

Furthermore, inspired by the highly stretchable sericin-bundled silk, Li et al. developed a super-fiber membrane with omnidirectional superelasticity, permeability, and superhydrophobicity (SPSM) through the synchronized electrospinning of styrene-isoprene-styrene (SIS) block copolymer and the electrostatic spraying of fluorinated SiO_2_ nanoparticles (see Figure 4e) [143]. The SPSM-based self-cleaning single-electrode stretchable TENG (STENG) was demonstrated by Li et al. to be adaptable to tapping, stretching, bending, and humidity, enabling its use as a breathable self-powered sensor for material identification and hand posture monitoring. Zhang and Yuan, inspired by animal structures, designed a triboelectric soft actuator (TEG-SA) that generates electricity through strain mismatch when heated [144]. The TEG-SA, driven by an LCE film, contracts upon heating and stores internal stress, which causes bending and makes the triboelectric layer contract to produce an electrical signal (see Figure 4f). This device is suitable for temperature-sensitive switches, with signals convertible into sound, light, or other forms for biomimetic effects.

In conclusion, integrating various biomimetic structures into TENGs can endow these devices with multiple functionalities, thus expanding their use across a variety of applications. These designs, inspired by nature, not only boost the output performance of TENGs but also bring in new features such as breathability, biodegradability, and responsiveness to environmental changes, which are crucial for sophisticated applications in areas like wearable electronics, environmental monitoring, and biomedical engineering. Nonetheless, these biomimetic structures have their limitations: the complexity of natural structures makes them difficult to replicate with precision, and even minor design variations can lead to significant impacts on device performance. Moreover, the integration of these structures might limit design flexibility or restrict the integration with other technologies.

### 4.3. Multilayer Structure

In the realm of electrospun nanofiber-based triboelectric nanogenerators (TENGs), the utilization of multilayer structures has demonstrated substantial potential for enhancing performance. Multilayer structure design entails incorporating multiple functional layers into TENGs, optimizing the triboelectric effects of nanofibers and elevating overall energy conversion efficiency [145,146]. These layers may involve different material combinations, diverse nanofiber arrangements, or stacked structures to achieve higher charge density and more stable electrical performance [59,147,148].

**Figure 5:**
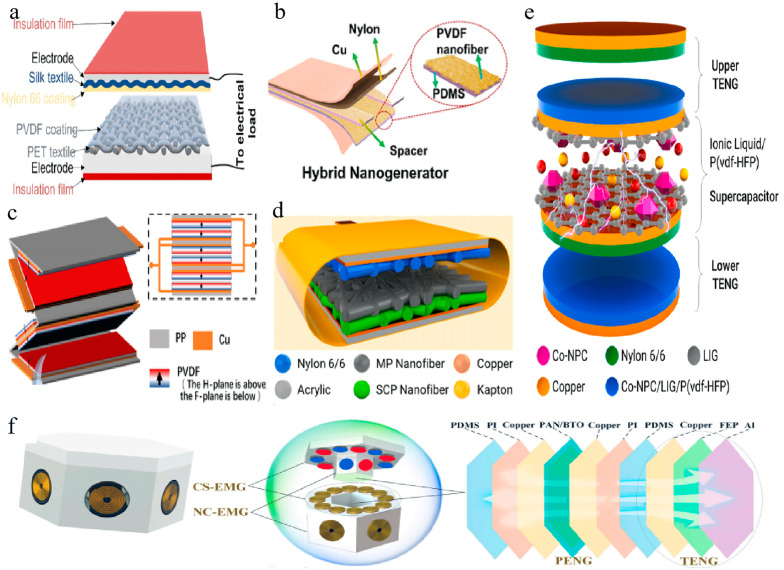
Multilayer structure. (**a**) Schematic representation of the TENG construction [149]. (**b**) Structure of PT-NG [152]. (**c**) Schematic diagram of the hybrid generator [155]. (**d**) Schematic diagram of the double-layer nanofibrous TENG [156]. (**e**) Schematic illustration showing the layer-by-layer structure of the self-charging SPC [157]. (**f**) Structural model diagram of the MS-CES [158]. All essential copyrights and permissions received.

For instance, Bairagi et al. developed a multilayer TENG structure by improving the positive triboelectric performance of silk with electrospun nylon 66 nanofibers and enhancing the negative triboelectric performance of PET with a PVDF coating (see Figure 5a) [149]. This approach achieved approximately 17-fold and 16-fold increases in output voltage and short-circuit current density, rising from 5.85 V to 100 V and from 1.6 mA/m^2^ to 24.5 mA/m^2^, respectively. The maximum power density reached 280 mW/m^2^ under a 4 MΩ resistance. In another study, Wang et al. proposed an enhanced electrospun fiber-based triboelectric nanogenerator (EF-TENG) featuring breathable antimicrobial electrodes and an electrostatic enhancement layer [150]. This was accomplished by integrating AgNW as electrodes and electrospun PS nanofibers as the charge storage layer. The TENG achieved a high output voltage of 200 V and a current density of 70 mA/m^2^ over a working area of 90 mm^2^, and could charge an economic capacitor from 1 μF to 2 V within just 40 s.

The hybrid piezoelectric/triboelectric structure is a common multilayer configuration in electrospun fiber-based TENGs, combining two types of energy harvesting mechanisms to enhance the overall output of the nanogenerator [32,44,55,151]. Chen et al. embedded electrospun PVDF nanofibers directly onto the surface of a flexible PDMS film to create a PVDF-PDMS composite membrane, which was then used to fabricate a sandwich-structured piezoelectric/triboelectric hybrid nanogenerator (PT-NG) (see Figure 5b) [152]. When a periodic external force of 10 N was applied to the PT-NG (1.33 × 1.33 cm^2^), it exhibited an open-circuit voltage output of 88 V and generated a maximum power density of 286 mW/m^2^ at a 25 MΩ load, significantly higher than that of individual TENG (136.72 mW/m^2^) and PENG devices (40.53 mW/m^2^). Additionally, the nanofiber structure of the composite membrane remained well preserved after 5000 test cycles, with the PT-NG device maintaining stable output performance. Moreover, Kumar et al. synthesized P (VDF-TrFE)/TiO_2_ nanocomposites via electrospinning and combined them with PDMS films to create a piezoelectric/triboelectric hybrid nanogenerator [153]. The hybrid nanogenerator generated a current of 5.36 μA when a 10 MΩ resistor was used with a voltage of 52 V.

Furthermore, some researchers have enhanced nanogenerator performance by assembling multiple layers of the same type of fibers [154]. Wang et al. prepared PVDF nanofiber membranes using electrospinning and employed a head-to-head parallel assembly approach to enhance electrical output (see Figure 5c) [155]. They also investigated the individual contributions and synergistic effects of triboelectricity and piezoelectricity in multilayer generators. The designed hybrid nanogenerator device achieved a maximum open-circuit voltage, short-circuit current, and charge value of 150 V, 7 μA, and 100 nC, respectively. Furthermore, by adopting the head-to-head parallel assembly method for the electrospun poly (vinylidene fluoride-co-trifluoroethylene) fiber membranes, the output performance was approximately four times higher than that obtained through conventional series connection.

In addition to the mentioned multilayer structures, some special multilayer structures also exist. For instance, Shrestha et al. designed a dual-layer nanofiber TENG material consisting of MXene/P (VDF-TrFE) as the charge generation layer and silicon oxide/Co-nanoporous carbon/P (VDF-TrFE) as the charge capture layer (see Figure 5d) [156]. This material, fabricated using a simple electrospinning process, exhibited twice the current density and surface potential compared to a single-layer nanofiber TENG, thanks to the charge capture layer. Additionally, the TENG with nylon 6/6 nanofibers as the positive triboelectric layer could provide a power density of 19 W/m^2^, demonstrating exceptional output performance compared to state-of-the-art products. Shrestha et al. also designed a self-charging supercapacitor (SPC), wherein the energy generated by the TENG was stored through a “triboelectrochemical mechanism”, without the need for power management or rectification circuits [157]. The self-charging SPC consisted of two layers of TENG and one SPC, all integrated into a single device with the TENG (see Figure 5e). This setup could generate 2.5 mW of power, successfully charging the SPC and reaching a maximum voltage of 210 mV within 9 s. Furthermore, Huang et al. proposed an innovative multilayer structure called a dual-mode electromagnetic/triboelectric/piezoelectric multifunctional self-charging energy system (MS-CES) [158]. This system integrates two different operating modes: contact-separation (CS-EMG) and non-contact (NC-EMG) electromagnetic generators, as well as triboelectric nanogenerators (TENGs) and piezoelectric nanogenerators (PENGs) (see Figure 5f). Each MS-CES unit demonstrates output performance across different vibration frequencies and amplitudes. The units work synergistically through a compact mechanical structure, which improves space utilization, shortens charging time, and achieves high-voltage output for more efficient energy storage.

The design of multilayer structures in TENGs enhances performance by increasing the frictional area and charge accumulation space, and by integrating various power generation methods like triboelectric, piezoelectric, and electromagnetic effects. However, this complexity introduces challenges such as ensuring compatibility between layers of different materials without compromising performance, which could lead to delamination or efficiency drops due to charge leakage. Moreover, optimizing each layer’s design is a meticulous process that requires a thorough understanding of material interactions, and adding layers might introduce mechanical weaknesses at interfaces, risking failure under stress or wear over time.

In summary, structural design is paramount in optimizing the performance of electrospun fiber-based TENGs. The fine-tuning of fiber structure, which includes fiber diameter, length, and arrangement, significantly enhances surface area and frictional contact, thereby improving charge generation and transfer efficiency. Incorporating biomimetic structures, such as blades or human skin patterns, not only boosts the energy conversion efficiency but also imparts TENGs with distinctive properties. Additionally, the adoption of multilayer configurations increases both the contact and friction area, offering more space for charge accumulation, which, in turn, elevates energy density and facilitates the integration of various power generation methods, enhancing overall performance. However, current structural designs fall short in meeting the full spectrum of requirements for TENGs, particularly in terms of durability, practical application, and ease of manufacturing. To unlock the full potential of TENGs, further innovation and research are imperative to overcome these limitations.

## 5. Applications

The integration of TENGs made from electrospun nanofibers into specific applications offers significant potential to improve their performance and expand their practical use. These nanofibers enable efficient energy conversion and open up a wide range of functional applications due to their adaptable structures. This integration plays a key role in advancing self-powered devices and holds great promise for environmental energy harvesting and wearable technologies.

### 5.1. Self-Powered Devices

Electrospun nanofiber-based TENGs offer significant benefits for self-powered devices [138,159]. These devices efficiently convert ambient mechanical energy, such as vibrations and pressure, into electrical energy, operating independently of external power sources [63,74,160,161]. This autonomy improves the durability and reliability of self-powered sensors, thereby lowering maintenance expenses [162,163,164]. Additionally, the flexibility of nanofibers enables TENGs to be effortlessly integrated into a variety of self-powered applications, fostering energy independence and contributing to sustainability efforts.

#### 5.1.1. Humidity Sensor

**Figure 6:**
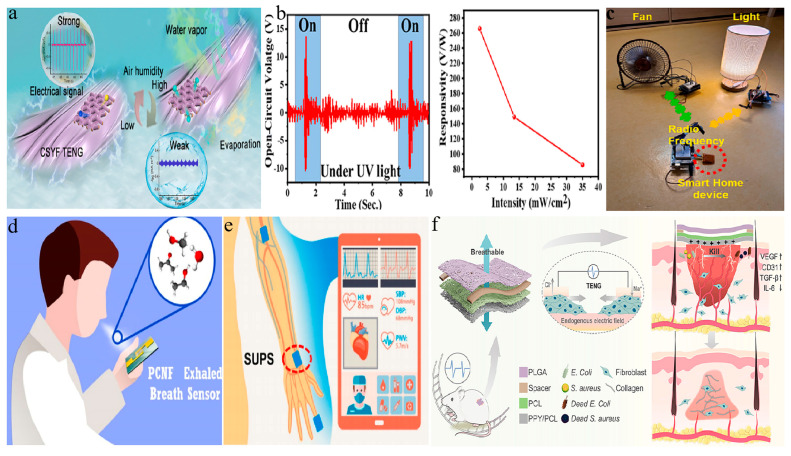
Self-powered devices based on electrospun nanofiber TENGs. (**a**) Schematic illustration of CSYF TENG as a self-powered humidity sensor [167]. (**b**) Output performance of STENG as visible-blind UV photodetector [169]. (**c**) A real-time smart home control system using an MOF/PVDF (MPVDF) NF-based TENG device (red circle marks the MPVDF NF-based TENG) [170]. (**d**) Schematic of a natural human breath test [171]. (**e**) Illustration of the integration of SUPS for noninvasive multi-indicator cardiovascular monitoring (red circle marks the SUPS) [172]. (**f**) Structure diagram of self-powered TENG and its principle diagram in wound healing (large grey arrow points to the position of TENG, indicating its location in the whole system; small grey arrows represent the healing of wounds from both sides, approaching towards the middle, showing the direction and process of wound healing) [173]. All essential copyrights and permissions received.

A self-powered device based on an electrospun nanofiber TENG can function as a humidity sensor for environmental humidity detection [165,166]. This innovative integration not only achieves the real-time and accurate monitoring of environmental humidity, but also eliminates the dependence on external power sources, significantly enhancing the device’s self-sufficiency and portability. For example, Zhou et al. developed a waterproof and breathable fabric-based TENG using nano/micro-core sheath yarns (CSYF TENG) with a micro/nano radial expansion fiber structure, showcasing excellent humidity management performance (see Figure 6a) [167]. Mathew et al. designed a self-powered micro-humidity sensor (SMHS) system based on titania nanocrystals (TiO_2_ NCs) embedded in electrospun nylon 6,6 nanocomposite films, which demonstrated excellent performance with a quick response time and recovery time [71]. Additionally, Ippili and colleagues developed a ZnAl–CO_3_-layered double hydroxide–PVDF composite film, which acted as a negative triboelectric material for a high-performance, flexible, and transparent TENG [168]. This film’s response to pressure and humidity enabled it to serve as an effective self-powered sensor for both conditions. It showed a pressure sensitivity of 13.07 V kPa^−1^ and a humidity response of 259.4%.

#### 5.1.2. Photodetection Devices

Integrating photovoltaic materials with TENGs can lead to the development of self-powered photodetection devices, used without an external power source to convert mechanical energy from the environment into electrical power [174,175]. This type of device has a broad range of potential applications, especially in display technology, wireless communication, and environmental monitoring [176,177]. In one study, Das et al. proposed a vanadium oxide (V_2_O_5_) hollow nanofiber-based supercapacitor-inspired TENG (STENG) as a self-powered visible-blind UV photodetector, demonstrating excellent performance as a self-powered visible-blind UV photodetector [169]. As depicted in Figure 6b, the STENG demonstrated significantly enhanced output performance under ultraviolet light irradiation, exhibiting high sensitivity even at low-power ultraviolet light conditions. Yan et al. designed a self-powered display system combining the PI nanofiber membrane-based TENG with electroluminescent fibers, demonstrating the diverse nature of the applications of these devices [177].

#### 5.1.3. Smart Home Systems

Furthermore, TENG self-powered devices based on electrospun nanofibers can widely be applied in smart home systems [7,30,68]. For example, Sohn et al. prepared PVDF nanofibers containing MOFs and combined them with aluminum electrodes to create a TENG with increased output power density [170]. This TENG was used as a self-powered trigger sensor in smart home devices, wirelessly operating various electronic devices (see Figure 6c). Additionally, Pandey et al. designed a high-performance triboelectric nanogenerator (NBP-TENG) based on novel Nafion-functionalized barium titanate nanoparticles (BaTiO_3_ NPs) and PVDF composite nanofibers, which can serve as a self-powered human–machine interface for home control systems [84]. Bhatta et al. developed a high-performance TENG using a 2D siloxene–polyvinylidene fluoride (S-PVDF) composite nanofibrous membrane [178]. When this TENG is combined with a highly sensitive capacitive pressure sensor (CPS), it serves as a self-powered pressure sensor ideal for identity recognition in smart home access control systems. Furthermore, Gao and colleagues designed a double-helix TENG using PVDF film as positive charge traps [179]. This TENG’s exceptional flexibility makes it an ideal triggering mechanism for smart home control systems.

#### 5.1.4. Personal Health Management

In the healthcare field, self-powered TENG devices offer a sustainable solution for personal health management [44,180,181]. These devices generate energy through minor contact and separation, enabling wireless and continuous monitoring [24,182,183]. Mohamadbeigi et al. developed a self-powered breath sensor using polyethylene oxide/copper oxide (PCNF) composite nanofibers and an FTO/Kapton triboelectric nanogenerator as the power source for detecting ethanol levels in exhaled breath (Figure 6d) [171]. The sensor holds promise as a potential biomarker for the early diagnosis of lung cancer. Xu et al. designed a self-powered ultraflexible pulse sensor (SUPS) based on a triboelectric nanogenerator for noninvasive multi-parameter cardiovascular monitoring [172], showcasing outstanding sensing performance and demonstrating the potential for noninvasive multi-parameter cardiovascular monitoring (see Figure 6e). Furthermore, TENGs based on electrospun nanofibers can serve as a self-powered device for wound treatment. Tang et al. designed a self-powered and intrinsic antibacterial patch based on a triboelectric nanogenerator (TENG) which provided remarkable outcomes for wound treatment (see Figure 6f) [173]. Cai and colleagues designed a PVDF film doped with BaTiO_3_, referred to as PVDF/BaTiO_3_, for the fabrication of a triboelectric nanogenerator (PB-TENG) [184]. This PB-TENG can serve as a self-powered sensor for diagnosing and monitoring muscle strains.

### 5.2. Environmental Energy Harvesting

With a growing emphasis on sustainable energy technologies, environmental energy harvesting has emerged as a significant research pursuit. The unique properties of nanofibers enable electrospun nanofiber-based TENGs to be particularly effective at capturing and converting the minute mechanical energy found in the environment. These attributes not only enhance energy conversion efficiency but also expand the potential applications of TENGs in the field of environmental energy harvesting. By carefully designing and optimizing the nanofiber structure, it is possible to effectively harvest various forms of environmental energy, including wind energy, acoustic energy, and water energy, thereby opening new frontiers in sustainable green energy utilization.

#### 5.2.1. Acoustic Energy Harvesting

**Figure 7:**
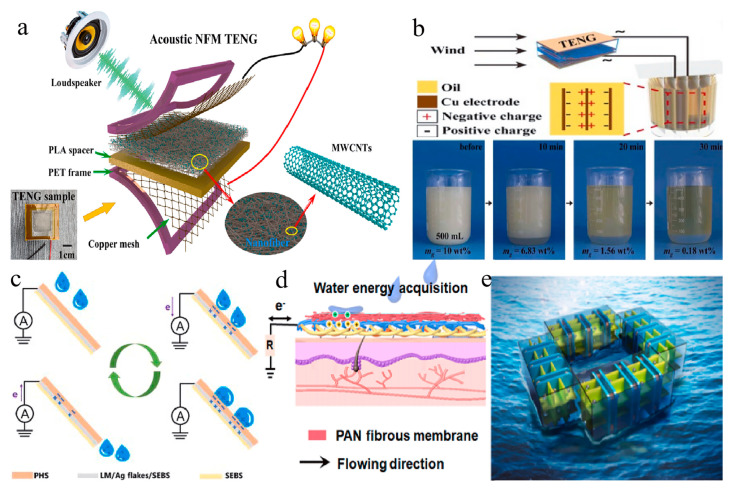
Environmental energy harvesting based on electrospun nanofiber TENGs (**a**) Acoustic NFM TENG (yellow arrow points to the overall structure of TENG; red arrow indicates a small part of the PLA layer and the MWCNTs within it; red line represents the wire) [191]. (**b**) Wind-driven TENG for W/O emulsion separation (red dash line marks the copper electrode and shows the charge distribution within it) [193]. (**c**) Water energy harvesting mechanism of the SNF-TENG (purple arrow shows the direction of charge movement) [194]. (**d**) When the rain droplets roll down the MWTT, triboelectric electricity is generated [195]. (**e**) Schematics of the G-TENG array for harvesting water wave energy [126]. All essential copyrights and permissions received.

In modern environments, acoustic energy, as a widely available form of energy, is gaining attention for its potential utilization. TENGs based on electrospun nanofibers provide novel opportunities for acoustic energy conversion [185,186,187]. These devices efficiently transduce the mechanical vibrations produced by sound waves into electrical energy [188,189,190]. For instance, Sun et al. developed a novel TENG based on a nanofiber membrane capable of harnessing sound energy from the environment [191]. The triboelectric nanogenerator exhibited remarkable performance, generating a maximum output voltage of 126.5 V and a current of 30.2 A with a power density of 2.25 W/m^2^ in response to sound wave stimulation at 116 dB and 200 Hz (see Figure 7a). Additionally, Xu et al. designed a laminated electrospun nanofiber TENG for self-powered real-time noise level monitoring, achieving a peak sensitivity of 53.6 V/Pa at a resonant frequency of 200 Hz [192].

#### 5.2.2. Wind Energy Harvesting

Concomitantly, wind energy, as a common, clean, and abundant resource, is gaining increasing research attention [196,197,198]. TENGs based on electrospun nanofibers effectively capture the minute vibrations caused by wind and convert them into electrical energy [199,200,201]. For example, researchers designed a TENG based on nylon and PVDF electrospun nanofibers to achieve emulsion separation by utilizing wind-generated electricity (see Figure 7b) [193]. Ren et al. also demonstrated a coaxial rotating standalone TENG for wind energy harvesting, which was integrated into a self-powered water splitting system for hydrogen production, utilizing ambient wind energy for electrolyzing water to produce hydrogen [202].

#### 5.2.3. Water Energy Harvesting

Combining water energy with TENGs based on electrospun nanofibers enables the efficient conversion of various forms of water energy, offering new pathways for sustainable energy development [203,204,205,206]. One example involves a self-locking, breathable, and waterproof nanofiber membrane-based TENG created using electrospinning and electrospraying techniques, which can harvest energy from flowing water. Additionally, multifunctional weather-responsive triboelectric textiles were designed to harvest energy from raindrops and convert it into electrical energy during rainfall events. Furthermore, a TENG grid (G-TENG) equipped with channels and pleated paper-based TENG was developed to convert water wave energy into electrical energy by utilizing impact force generated by water waves (see Figure 7c–e) [126,194,195].

These developments showcase the potential of TENGs based on electrospun nanofibers in various environmental energy harvesting applications, illuminating new pathways for sustainable energy utilization.

### 5.3. Wearable Devices

Among the diverse applications of electrospun nanofiber-based triboelectric nanogenerators (TENGs), wearable devices have particularly significant potential [42,93,207]. These devices have specialized energy supply requirements that necessitate efficient, flexible, and stable power solutions. TENG technology, characterized by its excellent energy conversion efficiency and flexibility, offers innovative possibilities for enhancing the design and functionality of wearable devices [208,209,210,211]. In this section, we explore the specific applications of TENGs in wearable devices, focusing on their uses in breath monitoring and human motion detection, along with the technological advancements that they promote. These applications not only expand the practical range of TENGs but also establish a robust foundation for the future development of smart wearable technology.

#### 5.3.1. Breath Monitoring

The high sensitivity and energy conversion capabilities of TENGs make them suitable for the real-time monitoring of users’ breathing patterns [88,212]. The flexible design of TENGs allows them to adhere to the skin or clothing, capturing subtle movements from breathing to generate voltage changes. This enables the precise detection of breathing rate, depth, and regularity, thus aiding in health management and disease prevention [90,213,214]. Currently, many researchers are integrating TENG technology based on electrospun nanofibers with masks to develop wearable electronic devices for monitoring breathing [152,215,216,217].

**Figure 8:**
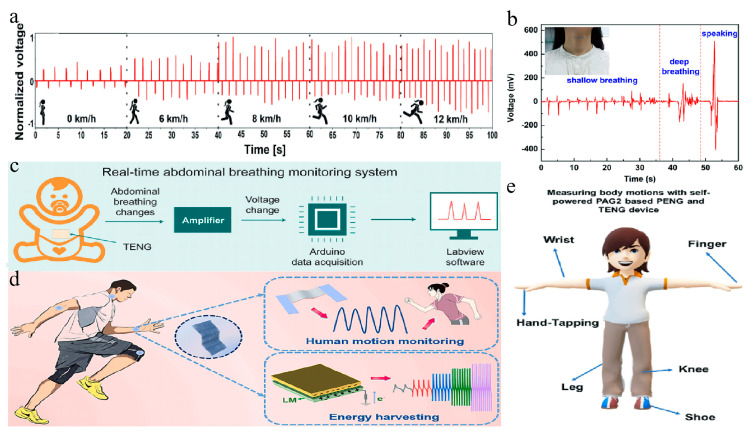
Wearable devices based on electrospun nanofiber TENGs (**a**) The TENG integrated into the mask is used to monitor breathing after walking or running at different speeds on a treadmill [218]. (**b**) The voltage changes in our device attached on throat muscle movement [219]. (**c**) Schematic diagram of the communication system for the real-time monitoring of abdominal respiratory status by the TENG sensor using a wired transmission device [107]. (**d**) The applications of ALTFM-based wearable electronics for human motion monitoring (pink arrows serve as pointers) [125]. (**e**) Human body movement recognition and detection using PENG and TENG devices based on PAG2-10 NFs fixed on different locations [220]. All essential copyrights and permissions received.

For example, He et al. designed a respiration monitoring TENG (RM-TENG) featuring a nanofiber membrane that functions as an intelligent, adjustable, self-powered mask filter [218]. This device uses PAN nanofiber mats as the positive triboelectric layer, with strategically positioned PVDF nanofiber mats to form deformable layers. Figure 8a illustrates the respiration signals detected during various physical activities, such as walking and running on a treadmill, highlighting the RM-TENG’s ability to effectively monitor the wearer’s breathing state through multiple indicators.

Lopez et al. developed an intelligent, low-cost, all-fabric TENG (AF-TENG) mask that employs ultra-high-molecular-weight polyethylene (UHMWPE) and cotton fabric as the negative and positive triboelectric layers, respectively [221]. This mask can monitor the patient’s breathing; if no signal is detected within a specified timeframe, a local alarm is triggered, providing crucial time for intervention.

Beyond masks, wearable devices utilizing electrospun nanofiber TENGs can also be affixed to the neck for breathing monitoring [140,222]. Jiang et al. innovatively integrated MXene nanosheets, known for their high negative triboelectricity and conductivity, with PVA to create a flexible all-electrospun TENG [219]. By employing PVA/MXene nanofiber films as the negative triboelectric layer and utilizing human skin as an electron donor, the TENG could effectively differentiate between shallow breathing, deep breathing, and speaking (Figure 8b). Additionally, TENGs can be attached to the abdomen to monitor breathing patterns. Hu et al. designed a fully fiber abdominal breathing monitoring sensor based on a TENG with a contact-separation mechanism, depicted in Figure 8c [107]. This sensor captures breathing signals, amplifies them, and transmits the data to a computer for real-time monitoring and analysis through Arduino and labview interfaces.

#### 5.3.2. Human Motion Detection

In addition to breath monitoring, human motion recognition is a critical application area for wearable devices. The introduction of TENGs based on electrospun nanofibers facilitates the accurate monitoring of users’ motion behaviors by efficiently capturing and converting subtle movements into electrical energy [223,224,225]. This capability enhances the precision of motion tracking, improving the overall user experience [226,227,228,229].

Li et al. proposed a flexible electronic skin (e-skin) with an aligned wavy structure capable of dual-mode strain and triboelectric sensing [125]. This e-skin is based on a composite material of aligned LM/TPU fiber mats, fabricated through electrospinning, coating, and pre-stretching techniques. As shown in Figure 8d, the device can be applied to various body parts for effective human motion recognition.

Furthermore, Wang et al. designed an ultra-light, breathable, sandwich-structured nanofiber composite composed of two PI nanofiber layers with embedded silver nanoparticles (AgNPs) and a conductive carbon nanotube (CNT) layer [230]. Enhanced triboelectric performance and self-sterilization capabilities were achieved through in situ reduction and electrospinning processes. This dual-channel or quad-channel wearable wireless monitoring system is capable of supporting gesture recognition.

Gunasekhar et al. investigated the piezoelectric and triboelectric properties of second-generation aromatic hyperbranched polymer (Ar.HBP-G2), synthesized with pentaerythritol as the core and bisphenol acid as the monomer [220]. By adjusting the Ar.HBP-G2 ratio blended with PVDF, they examined the electrical performance and mechanical stability of electrospun nanofibers used in PENG/TENG active layers. The optimized PENG/TENG was attached to different body parts during various movements, such as finger bending and knee motions, demonstrating varying voltage outputs based on the location of attachment (Figure 8e).

In addition to the previously mentioned TENG fabrication methods for motion detection, Zhang and colleagues created a MXene/PVDF-HFP composite fiber membrane [231]. This membrane was then assembled into a TENG using a TPU module. The resulting TENG serves as a self-powered motion sensor and wearable device, enabling human motion monitoring. Furthermore, it integrates with mobile applications to facilitate early screening for anterior cruciate ligament (ACL) injuries.

In conclusion, the integration of electrospun nanofiber-based TENGs into various applications, including self-powered devices, environmental energy harvesting, and wearable technologies, demonstrates their remarkable versatility and potential to revolutionize energy harvesting and utilization. Their ability to efficiently convert mechanical energy into electrical energy, combined with their adaptability, positions these nanofibers at the forefront of advancing sustainable energy solutions and wearable electronics, promising a future where energy autonomy and environmental sustainability are seamlessly woven into daily life. Nonetheless, the challenge lies in translating these advancements into practical, industrially viable solutions, highlighting the need for further research and development to bridge the gap between laboratory success and real-world application.

## 6. Summary and Conclusions

In conclusion, TENGs that incorporate electrospun nanofibers offer significant advantages in terms of energy conversion efficiency, flexibility, and a broad spectrum of applications. High-performance polymers such as PVDF and its copolymers, PA (nylon), TPU, and PAN have been extensively utilized, significantly improving the triboelectric performance and stability of TENGs. Structural design optimizations, including fiber structures, bionic structures, and multilayer configurations, have effectively enhanced the energy output and usability performance of TENGs, presenting broad application prospects in self-powered devices, environmental energy harvesting, and wearable technology.

Despite the significant advantages, the current research faces several challenges:

Efficiency: Although electrospun nanofibers have significantly improved the energy conversion efficiency of TENGs, further advancements are necessary. Key areas for development include optimizing material composition, refining the physical structure, and enhancing fabrication techniques to boost triboelectric performance, improve nanofiber design, and ensure consistently high efficiency across diverse environmental conditions, ultimately aiming for higher energy density and conversion efficiency.

Stability: The prolonged operation of TENGs can result in material aging and a subsequent decline in performance, highlighting the need for research into more durable materials, advanced nanofiber coatings, and resilient structural designs. Rigorous long-term testing under real-world conditions is critical to validate the reliability and practical utility of these devices.

Scalability: The transition from advanced laboratory-scale electrospinning techniques to commercial production poses challenges that include process consistency, quality control, and cost efficiency. Overcoming these challenges is pivotal for commercializing TENG technology and scaling up its market reach.

Moreover, future research on electrospun nanofiber-based TENGs may focus on the following areas:

New Material Exploration: Investigating novel polymers and composite materials to enhance triboelectric performance, increase stability, and improve overall efficiency in TENGs is crucial, involving the exploration of advanced polymers with high dielectric constants, nanocomposites that amplify the triboelectric effect through increased surface area, hybrid materials combining organic and inorganic properties, and functionalized materials tailored to enhance electron affinity or ion exchange capabilities, all aimed at pushing the boundaries of TENGs technology for broader and more efficient energy harvesting applications.

Structural Innovation: Designing new structures to enhance TENGs’ energy output and efficiency while retaining flexibility and durability is crucial for future advancements. This involves exploring novel designs, such as core–shell structures with different electrical properties for the core and shell to optimize charge separation and increase TENGs’ output, and self-healing structures that incorporate materials capable of autonomously repairing micro-damages to improve TENGs’ lifespan and reliability. These innovative designs aim not only to address current limitations in scalability and durability but also to push the boundaries of TENGs technology towards more versatile and efficient energy harvesting applications.

Integration and Smart Technology: By integrating TENGs technology with other energy harvesting systems like photovoltaic cells, piezoelectric generators, and thermoelectric devices, as well as with sensors and smart devices, we can develop comprehensive energy management solutions that not only increase the efficiency and adaptability of TENGs but also enable self-powered IoT networks, smart homes, wearables, and environmental monitoring systems, thereby broadening the scope and utility of TENGs in everyday applications.

Commercialization Advancement: Future research on electrospun nanofiber-based TENGs is poised to focus on achieving breakthroughs in commercial applications, particularly in wearable devices, smart home systems, and environmental monitoring. These advancements are essential for enabling large-scale production and market adoption by demonstrating the practical utility and economic viability of TENGs technology in real-world scenarios. Additionally, research efforts will aim to enhance the ease of manufacturing and production, which is crucial for making TENGs more commercially viable. By simplifying fabrication processes, improving scalability, and reducing costs, TENGs can be more readily adopted in various industries, thereby fostering sustainable energy solutions through their integration into diverse applications and systems.

In summary, TENGs technology based on electrospun nanofibers holds significant potential for improving energy conversion efficiency, stability, and scalability. Continued research and technological progress are anticipated to solidify TENGs’ critical role in advancing wearable technology, smart systems, and environmental monitoring, thereby fostering a more sustainable and energy-efficient future across various industries.

## Figures and Tables

**Table 1:** Positive triboelectric layer materials for nanofiber-based TENGs.

Materials	Electrode	Voltage	Current	Power	Application	Year	Reference
P: Mica/Nylon N: FEP	Al	400 V	40 μA	11.82 W/m^2^	Intelligent triboelectric wearable sensor	2024	[64]
P: Nylon/Na_2_SO_4_ N: FEP	Cu	494 V	30 μA	9.52 W/m^2^	Intelligent musical instrument	2024	[65]
P: Mica/Nylon N: PTFE	Al	629 V		5.36 mW/cm^2^	Energy harvesting	2024	[68]
P: EC/PA6 N: PVDF/MXene	Cu			290 mW/m^2^	Monitoring human movements as a self-powered sensor	2021	[66]
P: Nylon 6/MWCNT N: PTFE	Ag	105.7 V	10.55 μA	465 mW/m^2^	Self-powered sensors	2024	[70]
P: Nylon-6,6/TiO_2_ N: Cu	Cu		130 mA/m^2^	5 W/m^2^	Self-powered humidity sensors	2024	[71]
P: AgSbS_2_@ Nylon 6.6 N: PAN	Al	546 V		6.81 W/m^2^	Energy harvesting	2021	[67]
P: PA66/MWCNTs N: PVDF	Conductive fabric	142 V	15.5 μA	1.30 W/m^2^	Wearable electronics	2021	[62]
P: PLL/Nylon 11 N: Ecoflex	Al	137 V	3.4 μA		Self-powered wearable sensors	2023	[69]
P: EC/Nylon-11 N: PTFE/PVDF		212 V	18.5 μA	1.76 W/m^2^	Durable, wearable, and self-powered sensors	2023	[61]
P: poly-DADMAC/Nylon-11 N: PVDF-TrFE	Conductive fabrics	380 V	80 μA	7.6 W/m^2^	Self-powered sensors	2021	[63]
P: A-rGO/Nylon12 N: MoS_2_/Ecoflex	Al	451 V	13.3 μA	1.3 W/m^2^	Self-powered human–machine interfaces	2024	[72]
P: TPU/mica N: MXene/PVDF	Al	224 V	3.72 μA	1458 mW/m^2^	Body motion monitor	2023	[75]
P: PCA PNFMs N: TPU/CB BNFM	Conductive fabric	24.45 V			Biocompatibility, human motion detection	2023	[73]
P: EC/TPU/BTO N: PP	Cu	125.8 V	34.1 μA	1.68 W/m^2^	Self-powered sensors	2023	[74]
P: TPU N: FEP/MoS_2_		300 V	30 μA	4.2 W/m^2^	Wearable devices	2024	[16]
P: PAN/FCNT N: PVDF/PDMS/TiO_2_	Copper–nickel	2088 V		7.2 W/m^2^	Self-powered sensing fields	2022	[13]
P: ZIF-8/PAN N: PTFE	Al	178 V	7.5 μA	204.8 mW/m^2^	Energy harvesting	2024	[76]
P: PAN/B(OH)_3_ N: PVB	Al	200 V	45 μA	6.67 W/m^2^	Energy harvesting	2020	[77]
P: CNT/PAN N: PVP/EC	Al	960 V		14.6 W/m^2^	Energy harvesting	2022	[78]
P: CA NFs N: MXene NFs	Al	140 V	92 μA	1361 mW/m^2^	Self-powered sensors	2022	[79]
P: lignin/polycapro-lactone N: Teflon	Cu	96 V		157 mw/m^2^	Energy-harvesting	2023	[80]
P: PHB/PLCL N: ePTFE	Cu	460.63 V	25.49 mA/m^2^		Self-motivated sensor	2023	[81]
P: nickel-copper N: PAN/BaTiO_3_/MXene	Cu	11.3 V	300 nA	3.4 mW/m^2^	Self-powered wearable sensor	2024	[82]
P: GNP/TEOS/PVB N: PAN	Al	810 V	263 μA	20 W/m^2^	Wearable electronics	2021	[83]

Abbreviations: MWCNTs: multiwalled carbon nanotubes; poly-DADMAC: poly (vinylidenefluoride-cotrifluoroethylene); PP: Polypropylene; PVDF-TrFE: poly (diallyldimethylammonium chloride); A-rGO: amino-functionalized reduced graphene oxide; PLA: Polylactic acid; PDMS: polydimethylsiloxane; CS: chitosan; PCA PNFMs: PLA/CS/aloin porous nanofiber membrane; B (OH)_3_: Boric acid; BNFM: beaded nanofiber membrane, CB: carbon black; FEP: fluorinated ethylene propylene; ZIF-8: zeolitic imidazolate framework-8; PVB: Polyvinyl butyral; FCNT: functionalized carbon nanotube; TEOS: tetraethyl orthosilicate.

**Table 2:** Negative triboelectric layer materials for nanofiber-based TENGs.

Materials	Electrode	Voltage	Current	Power	Application	Year	Reference
P: Nylon-ZnO NWs N: PVDF-ZnO NWs	Al	330 V	10 μA	3.0 W/m^2^	Potent and sustainable power source for portable electronic devices	2020	[52]
P: Nylon-11 N: Co-NPC/PVDF	Al	710 V	210.96 mA/m^2^	19.71 W/m^2^	Energy harvesting and human motion monitoring.	2022	[94]
P: Nylon 6/6 N: PVDF/MXene	Cu	724 V	163.6 μA	11.213 W/m^2^	Self-powered foot motion sensor	2021	[95]
P: Al N: PVDF/HBP-G1	Al	65 V	1.76 μA		Energy harvesting	2024	[48]
P: Al N: PVDF/Si-HBP-G2	Al	130 V	3.5 μA	0.2 W/m^2^	Energy harvesting	2024	[47]
P: Al N: PVDF-Sep	Al	740 V	60 mA/m^2^	94.08 W/m ^2^	Energy harvesting	2023	[97]
P: Al N: PVDF/C_60_	Al			282 μW	Energy harvesting	2022	[98]
P: Al N: PVDF/GQD	Al			97 μW	Wearable mechanical energy harvesting	2019	[89]
P: Cu N: PVDF/MNS	Cu	163 V		585 μW/cm^2^	Energy harvesting	2023	[99]
P: Cu N: PVDF/PS	Cu	165.9 V	11.1 μA		Mask filter layer	2023	[100]
P: copper tape N: BaTiO_3_/PVDF	Cu	307 V	1.8 μA/cm^2^	1.12 mW/cm^2^	Energy harvesting performance and self-powered sensors	2023	[84]
P: natural seeds N: PVDF	ITO Al	126 V		324 mw/m^2^	Energy harvesting	2019	[85]
P: PVA/MWCNT N: PVDF	Cu	26.5 V	1.15 μA	4.57 W/m^2^	Self-powered sensors	2023	[86]
P: PVA N: PVDF/PZT	Cu	40 V	7 μA		Energy harvesting and self-powered sensors	2024	[101]
P: collagen/PVA/ Ag NW N: PVDF	Al	118 V	3.8 nA	21.06 mW/cm^2^	Mechanical energy harvesting	2022	[87]
P: TPU N: PVDF-CF	Ni-Cu	5.8 V			Identifying sleeping disorders and respiratory monitoring	2024	[88]
P: TPU N: P-Ar.HBP2	Ni-Cu	6.27 V			Polysomnographic and health monitoring	2023	[90]
P: TPU N: NiO/PVDF	Ni-Cu	252 V	7.3 μA	0.86 mW/m^2^	Energy harvesting and healthcare monitoring	2024	[51]
P: natural rubber N: BaTiO_3_/PVDF	Cu Ni	444 V	19.02 mA/m^2^		Harvest biomechanical energy	2022	[93]
P: PLLA N: MXene/MWCNTs-COOH/PVDF	Cu-Ni	124.23 V	54.48 μA	18.08 W/m^2^	Wearable portable electronic devices	2022	[91]
P: PTFE N: PVDF/KNN-ZS	Al	25 V	2.11 μA		Powering small-scale electronics	2022	[96]
P: JNA N: PVDF/PVP	Cu	155 V	6.20 μA		Energy harvesting	2021	[92]
P: Nylon-11 N: PVDF-TrFE/ MXene	Conductive fabrics	270 V	140 mA/m^2^	4.02 W/m^2^	Actuators and sensors	2021	[104]
P: Cu N: Bi_2_WO_6_/PVDF-TrEE	Cu Al	205 V	11.91 mA/m^2^		Self-powered sensors and smart electronics	2022	[106]
P: Nitrile N: BaTiO_3_: La/PVDF-TrEE	Al	245 V		2.52 W/m^2^	Energy harvesting and wireless power transmission	2023	[110]
P: PCL N: DIPAB/PVDF-TrFE	Conductive textile Cu	203.8 V		416.2 mW/m^2^	Energy harvesting	2024	[111]
P: SF N: PVDF-HFP	Ag	120 V	0.33 μA	40 μW/cm^2^	Wearable devices and energy systems	2024	[107]
P: skin, socks N: PVDF-HFP/ SiO_2_	LM-Ag-SEBS	114.5 V	4.9 μA	445 mW/m^2^	Self-powered electronic devices and wearables	2023	[108]
P: Kapton N: P3HT/PVDF-HFP	Al	78 V	7 μA	0.55 mW	Self-powered sensors	2022	[112]
P: TPU N: PVDF-HFP/LM		1680 V		24 W/m^2^	Energy harvesting	2021	[113]
P: PLA/TIO@ZIF N: conductive non-woven PLA fabrics	Conductive non-woven PLA fabrics	17.9 V	39.6 nA		Self-powered respiratory monitoring	2024	[17]
P: aPLLA N: PHB	Mg	45 V	9 μA		Energy harvesting	2023	[3]
P: silicone rubber N: modified-MXen-e/MoS_2_/CA	Cu	140 V		2975 mW/cm^2^	Distributed energy harvesting	2023	[114]
P: Al N: PI/CNT@BTO	Al	305 V	104 μA		Energy-harvesting and self-powered sensing systems	2024	[115]
P: PMMA N: JNM	Cu	353.5 V	22.4 μA		Electronic skin or wearable devices	2022	[116]

Abbreviations: PCL: poly (ε-caprolactone); PVA: polyvinyl alcohol; CF: cobalt ferrite (CF, CoFe_2_O_4_, a metal ferrite); GQD: graphene quantum dot; P-Ar.HBP2: PVDF/aromatic hyperbranched polyester of 2nd generation; Si-HBP-G2: second generation of silane-based hyperbranched polymer; MNS: mica nanosheet; Sep: sepiolite powder; PZT: lead zirconate titanate; JNA: Janus nanobelt array; Bi_2_WO_6_: bismuth tungstate; BaTiO_3_: La: La-doped BaTiO_3_; Kapton: polyimide; SEBS: styrene–ethylene–butylene–styrene; SF: silk fibroin; PLLA: poly (l-lactic acid); aPLLA: aligned PLLA; PI: polyimide; MWCNTs-COOH: two-dimensional MXenes and one-dimensional multiwalled carbon nanotubes.

## Data Availability

No new data were created or analyzed in this study. Data sharing is not applicable to this article.
